# Comparison of Post-Tonsillectomy Hemorrhage between Monopolar and Plasma Blade Techniques

**DOI:** 10.3390/jcm10102051

**Published:** 2021-05-11

**Authors:** Andy Wei-Ge Chen, Mu-Kuan Chen

**Affiliations:** Department of Otorhinolaryngology, Head and Neck Surgery, Changhua Christian Hospital, Changhua 500, Taiwan; 169277@cch.org.tw

**Keywords:** tonsillectomy, plasma blade, uvulopalatopharyngoplasty

## Abstract

The plasma blade is an innovative device that was recently introduced for performing tonsillectomy. While one of the benefits of the plasma blade is limited thermal damage, the effects of plasma blades on postoperative hemorrhage have not been thoroughly investigated. Patients who underwent tonsillectomy in our institution between January 2013 and September 2018 were retrospectively enrolled in the study. A total of 1214 patients were enrolled in the study, with 759 participants who underwent monopolar tonsillectomy and 455 participants who underwent plasma blade tonsillectomy. In total, 14 bleeding events occurred in the monopolar group, and 10 events occurred in the plasma blade group. The odds ratio for postoperative bleeding in the plasma blade group was 1.20 (95% CI 0.52 to 2.72). After adjusting for potential confounders, the adjusted odds ratio was 1.34 (95% CI 0.58 to 3.07). In conclusion, there is no significant difference in post-tonsillectomy hemorrhage rates between the traditional monopolar technique and plasma blade technique. Plasma blade tonsillectomy can be considered as safe as traditional monopolar tonsillectomy.

## 1. Introduction

Tonsillectomy is one of the most common procedures performed by otolaryngologists worldwide. Indications for surgery include chronic infection, upper airway obstruction, and suspected neoplasm [1]. Although most tonsillectomies are performed without complications, current studies demonstrate a 2% to 5% risk of post-tonsillectomy bleeding [2,3] in patients with normal coagulation function. Despite being extremely rare, life-threatening complications might occur due to post-tonsillectomy hemorrhages [4].

Numerous alternative devices have recently been introduced for tonsillectomy, including the coblation [5], microdebrider [6], Ligasure [7], and harmonic scalpel [8] devices. Several studies have pointed out that these modern devices may lower postoperative pain [5,6,7,8] and reduce operating time [7]. The PEAK plasma blade is a new device that uses radiofrequency energy to perform cuts and coagulation. The plasma blade has a lower average temperature (40–100 degrees Celsius) than the traditional monopolar blade (200–600 degrees Celsius), thus causing less thermal damage. Less thermal damage may lead to less tissue damage [9], decreased postoperative pain, and faster scar healing [9]. However, the lower temperature of the plasma blade may also cause difficulties with hemostasis during the operation. The impact of plasma blades on the postoperative hemorrhage rate [3] is an important but less thoroughly explored issue.

This study aimed to compare the post-tonsillectomy hemorrhage rate between the traditional monopolar technique and the plasma blade technique and therefore to investigate the safety of performing such procedure with plasma blade.

## 2. Materials and Methods

### 2.1. Trial Design

This was a single-institution, nonblinded, retrospective study performed in a tertiary medical center. The study was approved by the hospital’s Institutional Review Board (CCH IRB No.190206).

### 2.2. Patients

Patients who underwent tonsillectomy either due to chronic tonsillitis, obstructive sleep apnea, or suspected tonsil tumors in our medical center from 1 January 2013 to 30 September 2018 were enrolled in this study. Patients who underwent uvulopalatopharyngoplasty without tonsillectomy, for instance, those undergoing palatoplasty, were excluded from the study due to a very low rebleeding rate. Patients who underwent adenoidectomy without palatine tonsillectomy were also excluded due to similar reasons.

### 2.3. Methods

All patients were operated on under general anesthesia. Dedicated preoperative surveys were performed for all patients, including a chest X-ray, electrocardiogram, and blood survey for white blood cell count as well as coagulopathy.

The Dingman mouth gag, one of the most commonly used retractors, was chosen for exposing the operating field in our study. Initially, epinephrine at a concentration of 1/200,000 was injected into the tonsil fossa for bleeding control and hydrodissection. Blade No. 12 was used to perform the incision to enter the peritonsillar space. Blunt dissection was performed by the turbinate scissor at the superior pole of the tonsil.

In the monopolar group, monopolar electrocautery was used in the setting of 25 to remove the tonsil and obtain hemostasis. In the plasma blade group, the same procedure was performed by with the Medtronic Peak plasma blade on the setting of cutting 4 and coagulation 6. All patients were admitted after the operation. Antibiotics and steroids were not routinely given in the study.

Postoperative hemorrhage, defined as bleeding within 30 days of the operation that required surgical recheck, was chosen as the primary outcome.

### 2.4. Statistical Analyses

Data are expressed as the means and standard deviations. The differences between the groups were compared with independent sample t-tests. If the data were not normally distributed, the Mann–Whitney test was performed. Differences were considered to be significant when *p* < 0.05. Binary logistic regression was used to adjust the odds ratio. Statistical analyses were performed using the commercially available statistical software Medcalc, version 15.8 (MedCalc Software, Ostend, Belgium).

## 3. Results

Between 1 January 2013 and 30 September 2018, 1979 cases of tonsillectomy and uvulopalatopharyngoplasty were performed in the institution. 511 patients who underwent uvulopalatopharyngoplasty without tonsillectomy and 254 patients who underwent adenoidectomy only were excluded from the study. Ultimately, 1214 patients were enrolled in the study, with 759 participants who underwent monopolar tonsillectomy and 455 participants who underwent plasma blade tonsillectomy. The demographic variables are shown in Table 1.

The mean age of the monopolar group was older than that of the plasma blade group, 34 (95% CI for 33 to 36) versus 32 (95% CI for 29 to 34) years, respectively, with a *p*-value of 0.0002. Both groups had similar sex distributions. The body mass index in the monopolar group was 25.76 ± 7.87, which was significantly higher than the value of 24.53 ± 5.37 in the plasma blade group (*p*-value of 0.0013). There were no significant differences in the lab data, including white blood cell and platelet counts as well as prothrombin time.

**Figure 1:**
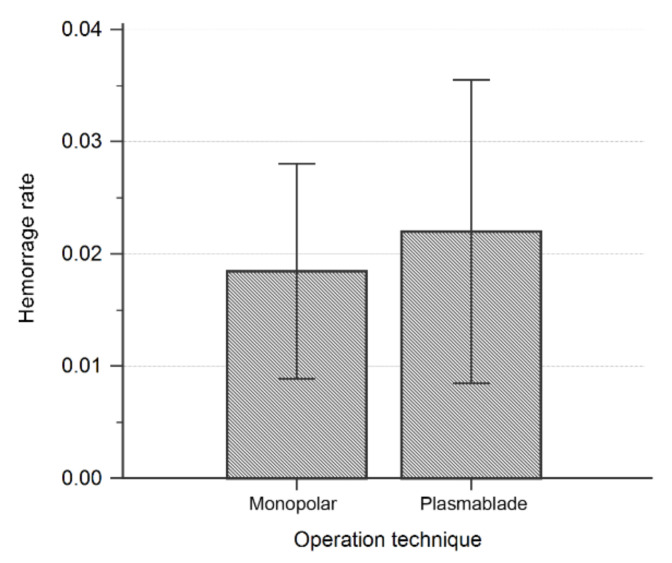
Postoperative hemorrhage rate.

The postoperative hemorrhage rate in both techniques is demonstrated in Figure 1 and Table 2. Within the population, 24 episodes of bleeding occurred. No participants experienced two episodes of hemorrhage. There were 14 episodes of bleeding among the 759 participants in the monopolar group and 10 episodes of bleeding among the 455 participants in the plasma blade group. No significant difference was noted between the two groups (*p* = 0.669).

The postoperative bleeding rate was 1.84% in the monopolar group and 2.20% in the plasma blade group. No statistical significance was noted (*p* = 0.669).

Table 2 demonstrates the results of the logistic regression model as crude and adjusted odds ratios. For analysis, the monopolar group was used as a reference. The odds ratio for postoperative bleeding in the plasma blade group was 1.20, with a 95% confidence interval from 0.52 to 2.72. After adjusting for potential confounders, such as age, sex, and body mass index, the adjusted odds ratio was 1.34, with a 95% confidence interval from 0.58 to 3.07. The 95% confidence interval of either the crude or adjusted odds ratio contains the value of 1, which suggests that there is no association between postoperative hemorrhage rates and the use of monopolar or plasma blade techniques.

### 3.1. Subgroup Analysis for Operation Indication

For subgroup analysis, 767 patients were operated on due to obstructive sleep apnea, and postoperative hemorrhage was noted in 16 patients. 447 patients were operated on due to chronic tonsillitis, and postoperative hemorrhage was noted in 8 patients. There was no significant difference between the two groups, with *p* = 0.721.

Among obstructive sleep apnea patients, 473 patients received monopolar tonsillectomy, and postoperative hemorrhage was noted in 10 patients. 294 patients received plasma blade tonsillectomy, and postoperative hemorrhage was noted in 6 patients. There was no significant difference in postoperative hemorrhage rate between the two techniques (*p* = 0.945) in the obstructive sleep apnea subgroup.

Among chronic tonsillitis patients, 286 patients received monopolar tonsillectomy, and postoperative hemorrhage was noted in 4 patients. 161 patients received plasma blade tonsillectomy, and postoperative hemorrhage was also noted in 4 patients. There was no significant difference in postoperative hemorrhage rate between two the techniques (*p* = 0.406) in the chronic tonsillitis subgroup.

### 3.2. Subgroup Analysis for Palate Treatments

For subgroup analysis, 530 patients received tonsillectomy without uvulopalatopharyngoplasty, and postoperative hemorrhage was noted in 8 patients. 684 patients received tonsillectomy with uvulopalatopharyngoplasty, and postoperative hemorrhage was noted in 16 patients. There was no significant difference between two groups, with *p* = 0.303.

In the tonsillectomy without uvulopalatopharyngoplasty subgroup, 312 patients received monopolar tonsillectomy, and postoperative hemorrhage was noted in 4 patients. 218 patients received plasma blade tonsillectomy, and postoperative hemorrhage was also noted in 4 patients. There was no significant difference in postoperative hemorrhage rate between the two techniques (*p* = 0.608) in the tonsillectomy without uvulopalatopharyngoplasty subgroup.

In the tonsillectomy with uvulopalatopharyngoplasty subgroup, 447 patients received monopolar tonsillectomy, and postoperative hemorrhage was noted in 10 patients. 237 patients received plasma blade tonsillectomy, and postoperative hemorrhage was noted in 6 patients. There was no significant difference in postoperative hemorrhage rate between the two techniques (*p* = 0.809) in the tonsillectomy with uvulopalatopharyngoplasty subgroup.

### 3.3. Subgroup Analysis for Surgeon Differences

A total of 26 surgeons were involved in the study. There was no statistical significance in postoperative hemorrhage rate between different surgeons, with *p* = 0.220. The median operating experience of the surgeons was 20 years, ranging from 5 to 34 years. There was no statistical significance in postoperative hemorrhage rate due to years of operating experience, with *p* = 0.581. We were unable to compare the postoperative hemorrhage rates of different techniques between different surgeons or their operating experience due to the low postoperative hemorrhage rate.

## 4. Discussion

The main objective of the current retrospective study was to investigate the differences in postoperative hemorrhage rate between monopolar tonsillectomy and plasma blade tonsillectomy. The postoperative hemorrhage rate was slightly higher in the plasma blade group than in the monopolar group, 2.20% versus 1.84%, respectively. However, there was no significant difference in either the crude or adjusted odds ratio. The results of this study support the safety of using a plasma blade in tonsillectomy compared to the safety of traditional monopolar techniques.

Most studies comparing complications in tonsillectomy are based on the pediatric population. In a nationwide study conducted recently in Taiwan, Hsueh et al. [10] pointed out that children in adolescence (12 to 18 years old) suffer from a higher risk of postoperative bleeding and reoperation than younger children. Several other studies [3,11] supported that older children suffer from a higher rebleeding rate than younger children. Adult cases, on the other hand, are often associated with increased fibrosis that can lead to greater blood loss than the pediatric tonsillectomy cases [12]. Reasonably, more cauterization is often used in adult patients to control bleeding. Thermal injury caused by cauterization may lead to postoperative pain.

The PEAK plasma blade has recently been introduced for tonsillectomy. This tool creates a highly ionized plasma field around the electrode using the surrounding tissue electrolytes, allowing the blade to dissect tissue with limited collateral damage. The working temperature of the plasma blade, 40 to 100 degrees Celsius, is much lower than that of the traditional monopolar electrocautery, which is 200 to 600 degrees Celsius [3]. While it causes limited thermal damage [9], the plasma blade can also make it difficult to control bleeding during the operation [13]. However, a previous animal study performed by Loh et al. [9] demonstrated that the plasma blade incisions have three times stronger wound strength by six weeks than electrosurgical incisions. A similar study performed on human skin by Ruidiaz et al. [14] suggested that plasma blade incisions have a reduced inflammatory response and stronger scar burst strength than incisions made with electrosurgical instruments. In contrast, Yilmazer et al. [13] found that there are no differences in tonsillar fossa wound healing and complication rates between plasma blade and cold dissection tonsillectomy techniques in a small prospective, randomized controlled study. In our study, plasma blade tonsillectomy had a slightly higher postoperative bleeding rate than monopolar tonsillectomy, but the result was not statistically significant (2.20% versus 1.84%, with *p*-value of 0.669).

Postoperative bleeding is a known complication in tonsillectomy patients, with an incidence rate ranging from 0.9% to 20% [3]. Wide ranges were noted in different studies due to different situations and definitions. The readmission rate for rebleeding after pediatric tonsillectomy in Taiwan from 1997 to 2012 was 0.9% [10]. A meta-analysis performed in 2001 pointed out that the post-tonsillectomy bleeding rate was 3.3% in studies of patients with normal coagulation and 8.7% in studies of patients with abnormal coagulation [2]. While tonsillectomies are often arranged as outpatient procedures in Western countries, most tonsillectomies performed in Taiwan are performed as inpatient operations. A population-based survey conducted in Taiwan using the Taiwan National Health Insurance Research Database pointed out that 97 percent of pediatric tonsillectomy patients from 1997–2012 are admitted at least overnight [10]. The postoperative hemorrhage rate might vary between different countries since inpatient cases have close postoperative follow-ups and good accessibility for second checks in the operating room. On the other hand, the definition of postoperative hemorrhage differs between literatures. Only cases where patients required a second surgery in the operation room for hemostasis were defined as postoperative hemorrhage in this study. Strict criteria might be another reason for the lower postoperative hemorrhage rate in this study.

While surgical instruments might influence the postoperative hemorrhage rate, the surgery technique is important as well. A systemic review [15] revealed that intracapsular dissection tonsillectomy has a lower secondary postoperative hemorrhage rate than extracapsular techniques. All patients in our study received extracapsular dissection tonsillectomy. The differences between monopolar and plasma blade techniques in intracapsular dissection tonsillectomy remain an interesting topic. Further studies can be conducted in the future.

As previously described, our patients received tonsillectomy mostly under blunt dissection, but the tonsils were removed by monopolar or plasma blade. Strict cold tonsillectomy, which often involves ligation of the inferior tonsil or the use of a wire snare, may lead to different results. Analysis done from national register data in Sweden [16] found that cold dissection with hot hemostasis has a 2.8 times higher rate of late postoperative hemorrhage than cold dissection with cold hemostasis. A similar result was seen in another study done in Wales through their Surgical Instrument Surveillance Programme [17]. All techniques that employ the use of any grade of heat have greater adjusted odds of late postoperative hemorrhage than strict cold tonsillectomy.

There are several limitations of this study. First, this was a nonblinded retrospective study. The selection of monopolar or plasma blade tonsillectomy was up to the patient’s preference, and this may cause some bias between the two groups. Conducting a single-blinded randomized controlled trial may provide more evidence. Second, only postoperative bleeding patients requiring recheck or hemostasis in the operating room were defined as having an event. Patients with mild oozing who only required observation in the outpatient or emergency room were not counted as having a postoperative bleeding event. The complication rate might be underestimated in this situation. Finally, bleeding is a rare complication. Despite enrolling 1214 participants in the current study, there were only 24 postoperative bleeding events. Multicenter studies could be helpful for achieving a larger sample size, but the surgeons and surgical methods might vary between different institutions.

## 5. Conclusions

The current retrospective study enrolled 1214 patients who underwent tonsillectomy in a single institution. There was no significant difference in the post-tonsillectomy hemorrhage rate between the traditional monopolar technique and the plasma blade technique. Although larger multicenter studies are needed to confirm this result, plasma blade tonsillectomy may be considered as safe as the traditional monopolar tonsillectomy.

## Figures and Tables

**Table 1:** Patient demographics.

Characteristic	Total (*N* (%))	Monopolar [*N* (%)]	Plasma Blade (*N* (%))	*p*-Value
No. of patients	1214	759 (62.5%)	455 (37.5%)	
Age (Years)				
Median	34	34	32	0.0002
Interquartile range	28	27	31.5	
Range	2 to 81	2 to 79	3 to 81	
Gender				
Male	718 (59.1%)	461 (60.7%)	257 (56.5%)	0.145
Female	496 (40.9%)	298 (39.3%)	198 (43.5%)	
Body Mass Index	25.30 ± 7.06	25.76 ± 7.87	24.53 ± 5.37	0.0013
White blood count	7.91 ± 2.62	7.89 ± 2.49	7.92 ± 2.69	0.837
Platelet	258.83 ± 70.18	256.29 ± 72.82	263.11 ± 65.35	0.095
Prothrombin time	11.13 ± 1.21	11.10 ± 1.34	11.19 ± 0.88	0.260

**Table 2:** Rates of postoperative hemorrhage.

	Hemorrhage No./Total No. (%)	OR (95 CI%)		*p*-Value
		Crude	Adjusted	
Monopolar	14/759 (1.84%)	1.00 (reference)	1.00 (reference)	
Plasma blade	10/455 (2.20%)	1.20 (0.52–2.72)	1.34 (0.58–3.07)	0.491

## Data Availability

Data generated is included within the manuscript.
